# Supplementary material for: Identification and experimental validation of BMX as a crucial PANoptosis‑related gene for immune response in Spinal Cord Injury
Source: PLoS One. 2025 Jul 15;20(7):e0328002. doi: 10.1371/journal.pone.0328002 (PMC12262871; doi:10.1371/journal.pone.0328002)
Supplement: S1 File — (DOCX) [file pone.0328002.s002.docx]

**Gradient for biomarker molecular mass:**


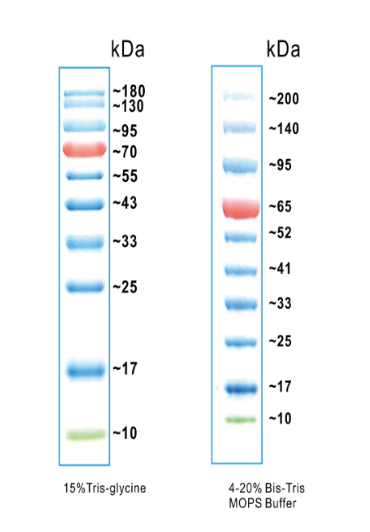


Raw bolt images for BMX (78 kDa):


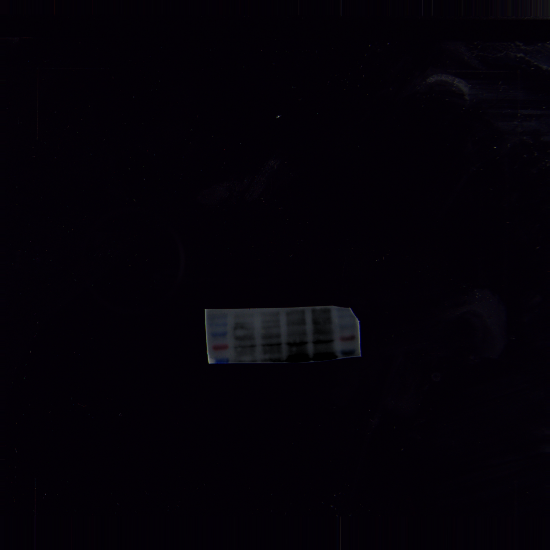


Raw bolt images for GAPDH (37 kDa):


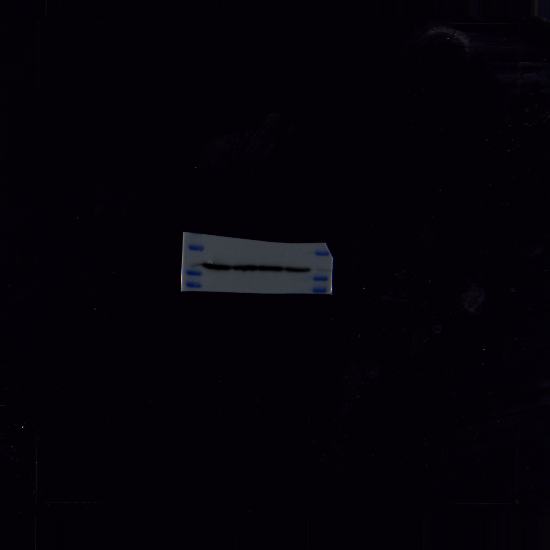


Raw images. Uncropped, unadjusted images of western blot.
