# Supplementary material for: Identification and experimental validation of BMX as a crucial PANoptosis‑related gene for immune response in Spinal Cord Injury
Source: PLoS One. 2025 Jul 15;20(7):e0328002. doi: 10.1371/journal.pone.0328002 (PMC12262871; doi:10.1371/journal.pone.0328002)
Supplement: Attachment — Submitted filename: Review to PanOptosis.pdf [file pone.0328002.s003.pdf]

The following are my review comments on the manuscript:

1. Introduction needs to re-structured as it should be stating the findings and background from literature to pave the way to the hypothesis, the necessity of the study conducted and neither state the processes of findings nor conclusions.
2. The reason behind selection of “GSE151371” needs to be ascertained in materials and method section.
3. “SCI rat model”, “Total RNA extraction and qPCR” are not in bold font whereas other sub-headings under materials and method section are. Please ensure consistency.
4. Please mention the cDNA synthesis kit catalogue.
5. “Figure 3C” should be in brackets in results section to maintain consistency with the rest of the manuscript.
6. In “fig4”, “Fig 10” formatting is suggested to be done as it has a lot of blank space.
7. Please illustrate briefly the “figure 5B”.
8. In “Fig6I”, “8A”, “Fig11” the upper limit of the Y-axis is missing/ uncapped
9. In “Fig 7A”, the box and whisker plot have the maximum and minimum caps missing.
10. The transcriptional activity quantification of BMX gene is appreciated. However, whether that cross talks to translational activity of the protein is crucial which at the moment is missing. So, my suggestion will be to include it.
11. The limitations of the study, specifically the need for validation of the bioinformatics findings by experimental work, needs to be better and clearly stated for every dataset.
