# Supplementary material for: Identification and experimental validation of BMX as a crucial PANoptosis‑related gene for immune response in Spinal Cord Injury
Source: PLoS One. 2025 Jul 15;20(7):e0328002. doi: 10.1371/journal.pone.0328002 (PMC12262871; doi:10.1371/journal.pone.0328002)
Supplement: Attachment — Submitted filename: Response to Reviewers.docx [file pone.0328002.s005.docx]

Response to Reviewer Comments

***Reviewer #1:***

***1.The authors used a Database of human patients some hours after spinal cord injury (SCI) to search for genes and/or pathways that could be important for this kind of lesion. I am only slightly familiar with bioinformatics, but I know that the results are as good as the database, and how it is analyzed and interpreted counts as much as the analysis itself.***

***First, the dataset you are based on is, as you cited, white blood cells of peripheral blood. Although the authors mention justified limitations for this manuscript, I still think some caveats should be addressed. As it is not a dataset with samples from the injured tissue (which, for the nature of the lesion, is very hard to accomplish), an important approach would be selecting cell markers to trace an inflammatory profile of the subjects that could correspond to the changes in the genes you show.***

***More importantly, I miss more in vivo data. The authors mention the heterogenicity of human samples. Here there was the chance to minimize such effects and to directly study how this lesion would affect individuals in a controlled environment.***

Response: In response to your concern about the dataset, we acknowledge that peripheral blood may not fully reflect the gene expression changes occurring in the injured tissue. While we did not specifically select cell markers to trace inflammatory profiles, we believe that the changes we observed in the peripheral blood reflect systemic immune responses that can be indicative of the inflammatory processes occurring after SCI. We plan to address this limitation in future studies by including more detailed analysis of specific cell populations involved in the inflammatory response.

Regarding your comment on the need for more in vivo data, we appreciate the suggestion and have now included additional in vivo experiments using SCI animal models. Specifically, we have conducted qPCR, Western blot and immunohistochemistry analyses on spinal cord tissue to validate the gene expression changes observed in the peripheral blood. These *in vivo* data help to directly assess the localized effects of SCI in a controlled environment and strengthen the findings from the peripheral blood analysis.

***2. When we perform animal experiments, we must use the maximum out of them for ethics sake. Here you brought only one PCR result from the spinal cord of the animals. The very fact that you studied the injured neural tissue in the animals and peripheral WBC in your dataset should be used as a caveat. To ease such discrepancy, you could run some RT-qPCR for inflammatory cell markers and try to relate that to what you find in the human peripheral WBC dataset you used. If the authors still have samples of the injured tissue for histology, immunohistochemistry or in situ hybridization could add better knowledge to your data and a visualization of the cell infiltration.***

Response: We acknowledge that our original manuscript included only one RT-qPCR result from the spinal cord tissue. In response to your suggestion, we have now included additional data from western blot (WB) and immunohistochemistry (IHC) experiments, both of which were performed on the spinal cord tissue from the SCI animal models. The WB results confirmed the protein expression changes observed in our PCR analysis, and the IHC experiments provided visualization of the protein localization, which allowed us to better understand the cellular distribution of inflammatory markers in the injured tissue.

While we did not conduct RT-qPCR analysis for inflammatory cell markers in this study, we agree that it would be beneficial to relate the findings from the spinal cord to the human peripheral WBC dataset. We plan to include such RT-qPCR analyses in future studies to further investigate the inflammatory response both locally and systemically.

Regarding your suggestion of histological analysis and in situ hybridization, we agree that these methods would provide deeper insights into the cellular infiltration in the injured tissue. However, the current manuscript focuses on the gene and protein expression levels. We acknowledge the value of these additional analyses and plan to incorporate them in future work to enhance the overall understanding of the injury-induced inflammation at the cellular and tissue levels.

***3. in vivo experiments you bring here are not a proper validation of BMX as the authors claim in the title. Better validation of the importance of a single gene to make it worth publication alone could include experimental groups either treated with blockers/antagonists of the gene transcript or knockout animals. The authors mention other genes with possible importance on the way to get to BMX, like the CASP5. An alternative to this issue would be also to check the top 3, top 5 genes that are altered in the bioinformatics analysis.***

Response: Thank you for your valuable suggestions. We understand your concern regarding our focus on *BMX*, and we would like to clarify the rationale behind this decision. In our initial machine learning-based analysis, *BMX* and *CASP5* emerged as the most prominent genes. While *CASP5* also showed interesting associations, we observed that *BMX* exhibited a stronger correlation with immune cell infiltration and was more consistently associated with the changes observed in both our bioinformatics analysis and subsequent validation. This stronger relationship with immune cell markers led us to prioritize *BMX* for further investigation, as it appeared to play a central role in the inflammatory processes we were studying.

However, we agree with your suggestion that expanding the analysis to include the top 5 genes would provide a more comprehensive understanding of the molecular mechanisms involved. We are currently exploring the other top genes identified in the bioinformatics analysis and plan to provide a more detailed discussion of these genes in future work. This will allow us to provide additional context for *BMX*’s role within a broader network of immune response-related genes.

We plan to further investigate these genes in subsequent experiments to better understand their contribution to the overall pathway.

***4. Nevertheless, I am also curious about why the authors chose to work with female rats. I suppose those were intact animals because you don’t mention any previous surgery. Was there any sort of estrus cycle control made to use the animals at the same time for all animals? Estrogen shows a protective effect on spinal cord injury (PMID: 36736846; 26461840), even therapeutically (PMID: 32680442; 12853305). This should be also raised as a limitation or a possible future direction, comparing males and females.***

Response: Female mice were chosen to maintain consistency in our experimental conditions and reduce variability that may arise from hormonal fluctuations in males. Additionally, female mice have a relatively shorter urethra, which presents a lower risk of urinary tract infections during the surgical procedure (PMID: 39104165; 40221710). Given the focus of our study on spinal cord injury, we aimed to minimize factors unrelated to the primary experimental variables, and this anatomical consideration played a role in our choice.

As you pointed out, the estrus cycle is an important factor that could affect experimental outcomes, particularly in studies involving spinal cord injury. Although we did not control for the estrus cycle in this study, we recognize that hormonal fluctuations could influence the results, and we will address this limitation in the revised manuscript. Furthermore, we agree with your suggestion to investigate the differences between males and females in future research, as this would provide valuable insights into the potential impact of sex on the injury response.

***Minor points:***

***- Gene names, scientific names, and Latin terms such as in vivo should be in italics.***

Response: Thank you for your valuable feedback. We appreciate your suggestion regarding the formatting of gene names, scientific names, and Latin terms. We acknowledge the importance of adhering to proper scientific conventions, and we will ensure that all gene names, scientific names, and Latin terms (such as *in vivo*) are italicized in the revised manuscript.

***- Supplementary Table 2 should be incorporated into the manuscript.***

Response: We appreciate your suggestion to incorporate Supplementary Table 2 into the manuscript as Table 1. We have now made the requested change and included it accordingly.

***Reviewer #2:***

***1.*** ***Introduction needs to re-structured as it should be stating the findings and background from literature to pave the way to the hypothesis, the necessity of the study conducted and neither state the processes of findings nor conclusions.***

Response: In response to your comment, we have restructured the introduction to clearly outline the background and findings from the existing literature, which lay the foundation for the hypothesis of our study. We have also ensured that the introduction now focuses on justifying the necessity of the study, without delving into the specific processes of the findings or premature conclusions. This restructuring should provide a clearer and more logical flow, leading the reader to understand the importance of our research and the gap it seeks to address.

***2. The reason behind selection of “GSE151371” needs to be ascertained in materials and method section.***

Response: We thank the reviewer for this insightful comment. Although our experimental model involves spinal cord tissue collected from injured rats, we selected the GSE151371 dataset—which contains RNA sequencing data from peripheral blood leukocytes of human SCI patients—as a complementary resource. This dataset was chosen because it offers transcriptomic profiles that reflect systemic immune and inflammatory responses after spinal cord injury, which are known to influence the progression of secondary damage.

Despite the difference in tissue origin, the GSE151371 dataset provides a clinically relevant perspective and allows us to identify potentially conserved molecular signatures between systemic and local responses. This cross-species, cross-tissue approach can enhance our understanding of key regulatory pathways involved in SCI.

In response to the reviewer’s suggestion, we have revised the Materials and Methods section to include a clear justification for selecting this dataset.

**3. “SCI rat model”, “Total RNA extraction and qPCR” are not in bold font whereas other subheadings under materials and method section are. Please ensure consistency.**

Response: In response to your comment regarding the inconsistency in the formatting of subheadings in the Materials and Methods section, we have made the necessary revisions. Specifically, we have ensured that "SCI rat model" and "Total RNA extraction and qPCR" are now in bold font, consistent with the other subheadings in the section.

We hope this change enhances the readability and uniformity of the manuscript. Thank you again for your valuable input.

**4. Please mention the cDNA synthesis kit catalogue.**

Response: In response to your suggestion, we have updated the manuscript to include the catalogue information for the cDNA synthesis kit used in our study. The specific catalogue number and the manufacturer’s details are now provided in the revised Materials and Methods section to ensure full transparency and reproducibility of the methods used.

Thank you again for your constructive feedback, which has helped improve the clarity and completeness of the manuscript.

***5. “Figure 3C” should be in brackets in results section to maintain consistency with the rest of the manuscript.***

Response: In response to your suggestion, we have updated the Results section to ensure that "Fig. 3C" is placed in brackets, in alignment with the formatting used for other figures throughout the manuscript. This change should now ensure consistency in the presentation of figure references.

***6. In “fig4”, “Fig 10” formatting is suggested to be done as it has a lot of blank space.***

Response: In response to your comment, we have carefully revised the layouts of these figures to minimize the excess blank space, ensuring that the figures are more compact and visually balanced. We believe this change will improve the overall presentation and clarity of the figures in the manuscript.

Thank you once again for your constructive feedback, which has greatly enhanced the quality of our manuscript.

***7. Please illustrate briefly the “figure 5B”.***

Response: We thank the reviewer for the helpful suggestion. As recommended, we have revised the Results section to include a brief explanation of Figure 5B. Specifically, we now clarify that Figure 5B presents a Venn diagram showing the overlap of the top 15 genes identified by five cytoHubba algorithms, with the intersection highlighting 10 robust hub genes. These genes are likely to play central roles in the regulatory network of spinal cord injury.

The corresponding text has been added to the revised manuscript to improve clarity and interpretation.

***8. In “Fig6I”, “8A”, “Fig11” the upper limit of the Y-axis is missing/ uncapped.***

Response: We appreciate the reviewer’s observation. In response, we have updated Figures 6I, 8A, and 11 to include the upper limits of the Y-axis. The axis limits have been capped to ensure clarity and to provide a more accurate representation of the data.

***9. In “Fig 7A”, the box and whisker plot have the maximum and minimum caps missing.***

Response: Thank you for your valuable feedback. We acknowledge the issue with Fig 7A, where the maximum and minimum caps were missing in the box and whisker plot. To address this and improve the clarity of the data presentation, we have updated the figure.

Specifically, we have replaced the box and whisker plot with a violin plot in Fig 7A. This change not only resolves the issue with the missing caps but also provides a more comprehensive view of the data distribution and variability. The violin plot combines the functionality of a box plot while also showing the data’s density distribution, offering more detailed statistical insights.

We appreciate your attention to the details of the figure, and we believe this modification will better convey the results of our study.

***10. The transcriptional activity quantification of BMX gene is appreciated. However, whether that cross talks to translational activity of the protein is crucial which at the moment is missing. So, my suggestion will be to include it.***

Response: In response to this suggestion, we have now included additional experiments to address the translational activity of BMX. Specifically, we have incorporated WB analysis and IHC experiments to evaluate the protein expression levels and localization of BMX. These additional data are now included in the revised figure and manuscript, providing a clearer connection between BMX gene expression and its translational activity.

We believe these additional experiments enhance the overall robustness of our study and provide a more complete understanding of BMX regulation. Thank you again for your constructive suggestion.

***11. The limitations of the study, specifically the need for validation of the bioinformatics findings by experimental work, needs to be better and clearly stated for every dataset.***

Response: In response, we have clearly stated the limitations of relying solely on bioinformatics data, particularly with respect to the GSE151371 dataset, which consists of transcriptomic data from peripheral blood leukocytes of SCI patients and healthy controls. While bioinformatics analyses provide important insights, we acknowledge the need for further experimental validation to confirm the relevance of these findings in vivo. We have conducted western blotting, qRT-PCR, and immunohistochemistry experiments in a rat SCI model to validate the expression of key genes such as BMX, and these experimental results are now included in the revised manuscript.

Additionally, we have acknowledged that the use of a single-source dataset and animal model may introduce some limitations, such as potential species-specific differences and the inability to fully represent the heterogeneity of SCI patients. We have highlighted the need for future research to incorporate diverse datasets from multiple sources and experimental methodologies to further validate and expand upon our findings.

We believe that these revisions address your concerns and strengthen the overall study. Thank you again for your constructive feedback.
