# Supplementary material for: Identification and experimental validation of BMX as a crucial PANoptosis‑related gene for immune response in Spinal Cord Injury
Source: PLoS One. 2025 Jul 15;20(7):e0328002. doi: 10.1371/journal.pone.0328002 (PMC12262871; doi:10.1371/journal.pone.0328002)
Supplement: Attachment — Submitted filename: Response to Reviewers.docx [file pone.0328002.s006.docx]

Response to Reviewer Comments

***Reviewer #1: I appreciate the authors’ thorough and satisfactory responses to my previous concerns. I have only a few minor corrections to suggest regarding Figure 11. In subpanel 11B, a required symbol is missing. Additionally, the figure legend does not describe the significance symbol used. Lastly, I noted a likely error in the description of the IHC, where a reference is made to the “Lamc1 protein,” which appears to be unrelated or included by mistake. Please address these issues in the revised manuscript.***

***Response to Reviewer #1:***

Response: We sincerely thank the reviewer for their careful evaluation of our manuscript and for acknowledging our thorough responses to previous concerns. We have addressed the minor issues raised as follows:

***Missing symbol in subpanel 11B of Figure 11:*** We have carefully reviewed Figure 11 and added the required symbol to subpanel 11B as suggested. The figure has been updated accordingly.

***Figure legend not describing the significance symbol:*** We have updated the figure legend to include a clear description of the significance symbol used in Figure 11. This change ensures that the figure legend fully corresponds with the content presented in the figure.

***Incorrect reference to “Lamc1 protein” in the IHC description:*** We appreciate the reviewer’s attention to this detail. After reviewing the manuscript, we found that the reference to the “Lamc1 protein” was an error. We have corrected this by changing it to “BMX protein” to accurately reflect the intended reference. The manuscript has been updated accordingly.
